# Mechanism of Silver Nanoparticles Action on Insect Pigmentation Reveals Intervention of Copper Homeostasis

**DOI:** 10.1371/journal.pone.0053186

**Published:** 2013-01-07

**Authors:** Najealicka Armstrong, Malaisamy Ramamoorthy, Delina Lyon, Kimberly Jones, Atanu Duttaroy

**Affiliations:** 1 Biology Department, Howard University, Washington, D.C., United States of America; 2 Department of Civil and Environmental Engineering, Howard University, Washington, D.C., United States of America; Queen Mary University of London, United Kingdom

## Abstract

Silver nanoparticles (AgNPs), like almost all nanoparticles, are potentially toxic beyond a certain concentration because the survival of the organism is compromised due to scores of pathophysiological abnormalities past that concentration. However, the mechanism of AgNP toxicity remains undetermined. Instead of applying a toxic dose, we attempted to monitor the effects of AgNPs at a nonlethal concentration on wild type *Drosophila melanogaster* by exposing them throughout their development. All adult flies raised in AgNP doped food showed that up to 50 mg/L concentration AgNP has no negative influence on median survival; however, these flies appeared uniformly lighter in body color due to the loss of melanin pigments in their cuticle. Additionally, fertility and vertical movement ability were compromised due to AgNP feeding. Determination of the amount of free ionic silver (Ag^+^) led us to claim that the observed biological effects have resulted from the AgNPs and not from Ag^+^. Biochemical analysis suggests that the activity of copper dependent enzymes, namely tyrosinase and Cu-Zn superoxide dismutase, are decreased significantly following the consumption of AgNPs, despite the constant level of copper present in the tissue. Consequently, we propose a mechanism whereby consumption of excess AgNPs in association with membrane bound copper transporter proteins cause sequestration of copper, thus creating a condition that resembles copper starvation. This model also explains the cuticular demelanization effect resulting from AgNP since tyrosinase activity is essential for melanin biosynthesis. Finally, we claim that Drosophila, an established genetic model system, can be well utilized for further understanding of the biological effects of nanoparticles.

## Introduction

For centuries, humans have exploited nanoparticles in small scale applications until the modern world has found engineered nanoparticles as tremendously useful in a diverse array of industrial products comprising of personal hygiene, clothing, food industry products, paints, cosmetics, pharmaceuticals, electronics and many more [1]–. Such large-scale use of nanoparticles obviously requires their massive disposal into the environment, which raises further concerns regarding the safety and fairness of such practice with regard to the environment and our ecosystems. Nanotoxicology includes comprehensive assessments of interactions between biological systems and specific nanoparticles based upon their delivery through various exposure routes since specific cellular mechanism(s) of the nanoparticles interaction with biological systems are mostly unknown.

The graveness of the environmental concerns have led to a rapid burst in studies to assess the toxicity of nanomaterials both *in vitro* and *in vivo* [5]. Exposure of cell cultures to various nanoparticles have been linked to DNA damage, oxidative stress, reduced photosynthesis, cytotoxicity, genotoxicity, apoptosis, necrosis, and aberrant mitochondrial function [6]–[11]. *In vivo* studies performed on a whole spectrum of organisms have been able to list variety of toxic effects from organismal exposure to nanoparticles such as developmental abnormalities in zebra fish embryos [12]–[13], declining lung function in vertebrates [14], reduced survival in fish and Daphina [15], interaction with immune cells in sea urchin [16], increased mutagenesis in Drosophila [17] and hepatic toxicity in mice [18]. In most studies though, the observed effects and pathologies were evident when the toxicity was acute. This is an important consideration since the environmental concentration of nanoparticles stay well below the toxic levels used in the laboratory.

AgNPs are especially popular because of their microbicidal properties [2]. With regard to the specific toxic effects of AgNPs, recent investigations highlighted that oxidative stress biomarkers like heat shock proteins, ER stress markers, lipid peroxidation, heme oxygenase, metallothionein and cell death activators like caspases are selectively upregulated under acute toxicity of AgNP [7], [19]–[21]. The current study is undertaken to evaluate the effects of silver nanoparticles (AgNPs) more on a mechanistic level, after exposing the fruit fly, *Drosophila melanogaster*, to a nonlethal dose of AgNPs over an extended period of time. A recent study [22] as well as this one confirmed that consumption of AgNP even at a nonlethal concentration caused cuticular demelanization in Drosophila. Probing into the biochemical processes helped us to elucidate a mechanism for the effect of AgNPs on melanization whereby the availability of extracellular silver cause sequestration of copper, without influencing the total copper uptake. Copper, unlike silver, is an essential component of living system [23]–[24]. We establish that AgNP accumulation affects the organism in a manner similar to copper starvation. Finally, we argue that Drosophila offers a very attractive model system for understanding the biological mechanisms of nanotoxicity with a plethora of biological and genomic reagents.

## Materials and Methods

### Source of Silver Nanoparticles (AgNP)

Citrate-stabilized silver nanoparticles (Cit-AgNP) were synthesized and characterized at Duke University in the Center for Environmental Implications of NanoTechnology (CEINT). The nanoparticles were synthesized with silver nitrate (1 mM) and sodium citrate (10 mM) as described earlier [25].

### Characterization of the Citrate-coated AgNP

The citrate-coated AgNPs synthesized in this work range in size from 1–50 nm, depending on the batch. For example, one batch of AgNPs in this study was 25.2±9.3 nm, as measured by TEM (Figure S1A). The concentration of silver in produced NP suspension is 100 mg/L, with some batch-to- batch variation. The absorption spectrum of a 10% dilution of the AgNP suspension displays a peak at 406 nm (Figure S1B). The produced AgNPs are elemental silver surrounded by approximately 0.65% w/w of a citrate coating [26].

### Fly Strains

All fly strains were obtained from the Drosophila Stock Center in Bloomington, Indiana. Complete genotypes of *Ctr1A* and *B* mutants are *w^1118^; Ctr1A^25^/FM6, w^1^* and *w^1118^; Ctr1B^3-4^/TM6B, Tb* respectively. For the *black* mutation, we used the *b^1^* allele. Unless otherwise mentioned, all fly cultures were maintained at 23±1°C in standard Drosophila medium that consisted of yeast, sugar, and corn meal with methyl paraben added as an antifungal agent.

### Dietary Administration of Silver Nanoparticles (AgNPs)

Two grams of dry Formula 4–24 *Drosophila* Instant Medium (Carolina Biological Supply Company, Burlington, NC) was placed in Drosophila culture vials and reconstituted with either 5 ml of sterile dH_2_O (vehicle control), or 125 µM of sodium citrate (citrate coating control), or 50 mg/L of citrate stabilized silver nanoparticles resulting in the following final concentration: ∼92 mg of sodium citrate per kilogram of food (mg/kg) and 125 mg of AgNPs per kilogram of food at room temperature. Wherever used, silver nitrate solution was prepared at the same concentration as Cit-AgNP although 50 mg/L of AgNO_3_ contains 31.74 mg/L of Ag ion. Before use, AgNP stock solution was vigorously shaken for one minute for complete dispersion of the particle, diluted to the required concentration, vortexed at full speed for 30 seconds, and immediately used for doping the culture media. Since the reconstituted medium is not a liquid medium, it reduces the likelihood of accelerated chemical transformations. Finally, it is apparent from the MSDS data sheet that Formula 4–24 is a non-toxic substance.

Most treatments were done using wild type *Drosophila melanogaster* (CantonS strain) unless specific genotypes are indicated. Males and gravid females were released on AgNP doped food or in control medium and allowed to lay eggs for 5 days. After the fifth day, the parents were discarded since enough eggs were laid during this time. Occasionally, a few drops of water were added to the culture to prevent dehydration. Drosophila, being a holometabolous insect, undergoes four developmental stages: embryo, larva, pupa, and adult stage. The larval stage represents the most voracious feeding stage where most AgNPs are consumed.

### Measurement of Tyrosinase Activity

Eighty-eight milligrams of flies were homogenized in lysis buffer containing 2% SDS, 1 mM of ascorbate, 1 mM bathocuproine disulfonate, complete protease inhibitor, and 50 mM Tris-Cl, pH 6.8 [27] using a hand held homogenizer. Lysates were pooled together in pairs, and 446 ml of pooled lysate was added to a substrate solution containing 1.5 mM L-Dopa, 4 mM 3-methyl-2-benzothiazolinone hydrazine (MBTH), and 2% N,N’ dimethylformamide [27]–[28] to a final volume of 1 ml. The substrate solution was kept at 37°C until the pooled lysate was added. Purified mushroom tyrosinase activity (Sigma-Aldrich, St. Louis, MO) (2 mg/ml) was used as a positive control. The change in absorbance at a wavelength of 505 nm (A_505_) was recorded every 15 seconds for up to five minutes. The activity of tyrosinase enzyme was determined and compared between the control and experimental flies.

### In Gel Superoxide Dismutase (SOD) Activity Assay

Twenty flies were homogenized in buffer containing 10 mM DTT, 4% glycerol, 0.1% Triton X-100 and complete protease inhibitor (Roche). Homogenates were electrophoresed on a 9% polyacrylamide gel under non-denaturing condition [29]. After the run, the gel was incubated in 1.23 mM nitroblue tetrazolium (NBT) salt prepared in 100 mM sodium phosphate buffer (pH 7.8) for 20 minutes in the dark and washed for five minutes in 100 mM sodium phosphate buffer (pH 7.8) to get rid of excess NBT. Then, the gel was soaked for 15 minutes in 28 mM of TEMED, 2.8×10^−2^ mM of riboflavin, and 100 mM sodium phosphate buffer (pH 7.8). The achromatic bands for copper-zinc SOD (Cu-ZnSOD) and manganese SOD (MnSOD) were visualized by exposing the gel for 15 minutes to the white fluorescent light source. SOD active areas appear as white bands in the gel amidst a blue background. Densitometry was performed using the Kodak Molecular Imaging Software version 4.5.

### Life Span, Fecundity and Pupal Eclosion

For the life span assay, young females were collected from each treatment cohort and placed onto standard medium within three days post eclosion. Females were transferred to fresh medium every three days, and the number of dead flies was recorded until all flies were deceased. Data analysis was done using the Log rank test for survival in Graph Pad Prism 4.03.

For fecundity analysis, freshly eclosed males and females from AgNP doped food were placed in regular medium (5 males: 5 females/vial) and several such cohorts were set up. To avoid crowding, parents were transferred every three to six days to fresh media and old cultures were kept for future progeny counting. The total number of adults emerged from each cohort is an indicator of parental fertility.

For the pupal eclosion assay, the total number of pupae and adults were tallied to determine the adult-to-pupae ratio after ingesting AgNP during development.

### Vertical Mobility Assay

Progeny that eclosed after the aforementioned treatment were collected up to six days post eclosion, and their vertical climbing behavior was monitored starting at eight days post eclosion. Ten flies were set up in four replicate vials per treatment. The number of flies that climbed four centimeters in 30 seconds in three consecutive repeats per vial was recorded every seven days for up to 35 days.

### Reverse Transcriptase Polymerase Chain Reaction

Total RNA was extracted from 30 flies with the RNeasy Plus mini kit (Qiagen). First strand synthesis was performed with the cloned avian myeloblastosis virus (AMV) first-strand cDNA synthesis kit (Invitrogen, Grand Island, NY), and the PCR reaction was carried out by denaturation at 94°C for one minute, annealing at 57°C for one minute, and extension at 72°C for two minutes. The following primers were used: *Ctr1A* forward (5′- TCGCACTTCAACAAACCTTG-3′), *Ctr1A* reverse (5′- ATTTGCTTTTT GGGGGAATC-3′), *Ctr1B* forward (5′-AGCACCTCAGCAGGTCTAGC-3′), *Ctr1B* reverse (5′-GCTTTAGATGAATACGCGTTG-3′), *Ctr1C* forward (5′-AATCGGGAACCCAGGTC TAC-3′), and *Ctr1C* reverse (5′-GCAAAAGACCTTGAGCTTGG-3′). The *RP49* gene was used as a standard internal control in all reactions.

### Graphite Furnace Atomic Absorption Spectrometry (GFAAS) for Silver and Copper Detection

One hundred milligrams of flies exposed to the vehicle control, citrate control, or 50 mg/L Cit-AgNP or AgNO_3_ were rinsed thoroughly with sterile distilled water and digested in concentrated nitric acid overnight at 80°C. Each sample was diluted with 0.2% nitric acid to a final nitric acid concentration of 2%. If the sample concentration was too high, the sample was further diluted with 2% nitric acid prior to repeating the silver measurement. Twenty microliters of each sample (in analytical triplicates) were used to evaluate the silver and copper levels using the PerkinElmer AAnalyst 800 Spectrometer. The absorbance values of silver and copper standard concentrations at 0 ppb, 20 ppb, 40 ppb, 60 ppb, 80 ppb, and 100 ppb, and were plotted as nonlinear through zero. Sample analysis was performed in triplicate using separate biological repeats and values were analyzed using the Student’s t-test.

### Silver Ion Selective Electrode (Ag^+^-ISE) Analysis

To quantify the Ag^+^ concentrations (in ppm) in the 50 mg/L Cit-AgNP solution in comparison to 50 mg/L AgNO_3_, the manual plotting method for Ag^+^ measurement was utilized according to the instructions provided for the Ag^+^/S^2-^ electrode (Fisher Scientific, Pittsburgh, PA) with minor modifications. The silver standards were prepared using serial dilutions at the following concentrations (in ppm): 1, 10, 100, and 1000. Forty milliliters of each standard plus 0.8 ml of the ionic strength adjustor (ISA) (5 M NaNO_3_) was used to generate a calibration curve. The potential (E) in millivolts (mV) was plotted against the logarithm of the concentration, and the curve was fitted with linear regression (R^2^ = 0.995) in Microsoft Excel. The linear equation provided was used to calculate the Ag^+^ concentration in the sample solutions. The samples and ISA were prepared at the same volume as mentioned above to measure the Ag^+^ in solution. All readings were performed in triplicate. Statistical analysis was performed using the Student’s t-test.

## Results

### Dietary Administration of AgNP Causes Cuticular Demelanization in Drosophila

**Figure 1:**
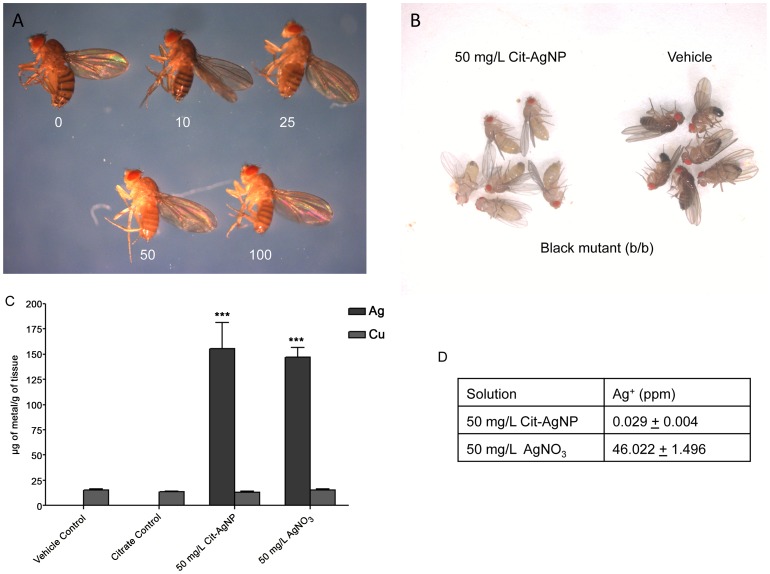
Dietary administration of AgNPs cause cuticular demelanization in Drosophila. (A) Flies are raised in AgNP doped food at various concentrations from the embryonic stage. When raised in 50 mg/L and above concentrations of nanoparticles, all adult flies (100%) appeared extremely lighter in body color with little or no melanin pigments left in their body. Since the eye color remains unchanged this suggests that AgNPs selectively interferes with the melanin pigmentation. (B) In Drosophila *black (b)* mutants accumulates excessive melanin pigments. Exposure of *black* flies to 50 mg/L AgNP effectively eliminates all melanin pigments from their body and the flies turned pale. (C) Sufficient accumulation of silver occurs in AgNP fed flies as determined by Atomic Absorption (AA) analysis. Flies fed with AgNO_3_ were used for comparison. (C) Measurement of free silver (Ag+) with ion specific electrodes confirmed that 50 mg/L AgNP solution generates ionic Ag^+^ at a negligible quantity when compared to AgNO_3_ solution in the same concentration. This led us to conclude that flies raised in AgNP doped food mostly consumed AgNPs and not free Ag^+^ to display the demelanization effect.

An earlier study [22] as well as this one reports that exposure to AgNP from early development causes demelanization of adult cuticle in *Drosophila*. As a result, all adult animals appeared totally bleached due to the lack of melanin pigments. In order to titrate the amount of AgNP required to cause the cuticular demelanization effect, the culture media was doped with increasing amounts of AgNPs up to 100 mg/L which led us to observe that from 50 mg/L onwards all adult flies appear uniformly bleached (Figure 1A). The demelanization phenocopy is not heritable although it happens uniformly in all adult animals, which indicates that the added AgNPs are dispersed well into the semisolid culture media. To further establish the observed effect of AgNPs on melanin pigmentation, AgNPs were administered to heavily melanized *black (b)* mutant flies in Drosophila medium, and they too appeared completely bleached after consuming AgNPs at 50 mg/L concentration (Figure 1B). Obviously, following dietary consumption of AgNP, Ag accumulation was spiked in demelanized flies as determined by atomic absorption (AA) analysis (Figure 1C) ensuring that AgNPs were actually ingested; hence, demelanization did not happen from cuticle contact. Ag is not an essential micronutrient in living organism, so the control flies (Figure 1C) failed to show any trace of Ag with AA analysis, however Ag concentration went up when flies were raised in either AgNP or AgNO_3_ doped media (Figure 1C).

### AgNP but not Ag^+^ are Potentially Responsible for Demelanization

While the effect of AgNPs on the melanization of the insect cuticle was compelling, we were still confronted with the question whether the observed demelanization effect resulted from AgNP ingestion or from ionic silver (Ag^+^) produced by AgNPs. As shown in Figure 1C, free Ag^+^ were formed only in negligible quantities in the 50 mg/L AgNP solution which was used to reconstitute the dry food. In contrast, at the same concentration AgNO_3_, which is readily soluble in water, forms ∼46X times more Ag^+^. Despite the formation of higher amounts of free Ag^+^ in AgNO_3_ solution, an earlier study has shown that a concentration of 0.001 grams of AgNO_3_ per 10 cc of regular cornmeal-molasses-agar food was not capable of causing any demelanization effect in flies; demelanization was observed at 0.005 grams of AgNO_3_ per 10 cc of food [30]. Finally, only a negligible reduction in cuticular pigmentation was reported when 10 mg/L AgNPs was used. All of the flies showed a significant reduction in body pigmentation following a 20 mg/L dose of AgNPs [22]. Therefore, the insignificant quantity of ionic Ag in the AgNP solution should not contribute to the cuticular demelanization effect.

### AgNP Consumption at Nonlethal Concentration Interferes with Other Biological Processes

**Figure 2:**
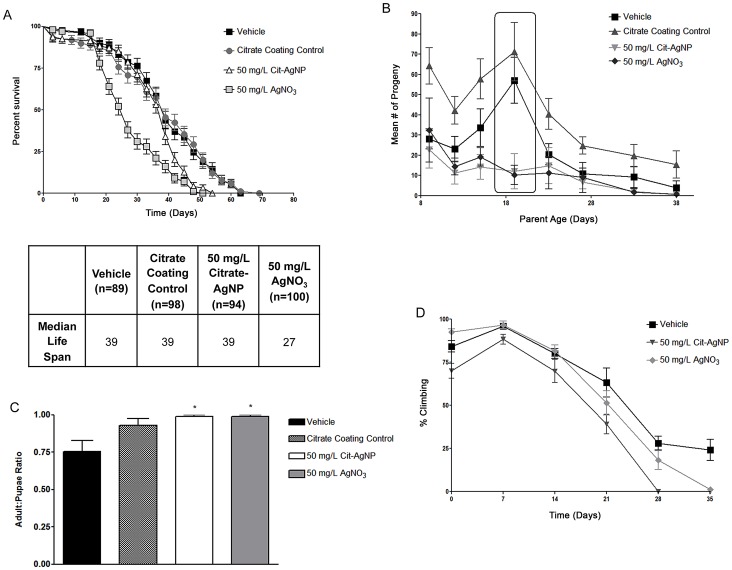
Loss of cuticular pigmentation does not alter the survival and metamorphosis, but female fertility and vertical climbing behaviors are affected. (A) Demelanized adult females live the same median life span as the controls, when adults were maintained in food without the AgNPs. A second control was set up with Na-citrate, which demonstrates, that citrate coating does not influence the survival. We therefore considered 50 mg/L as a nonlethal concentration for Drosophila. A significant drop in median life span was noted with AgNO_3_ feeding. (B) Number of progenies obtained from females raised in AgNP doped food was significantly lower than the controls especially during the peak egg-laying period, which is between 10–30 days (boxed). (C) Metamorphosis proceeds normally in demelanized flies. Percent pupae eclosed as adults was determined from #adult/#pupal ratios. Eclosion ratios are not significantly different between Cit-AgNP fed and unfed controls. * represents p<0.05. (D) AgNP fed flies are slow to climb the same vertical distance with respect to the unfed flies, and this effect was evident starting from a very young age which is sustained throughout life. Climbing performance was more seriously impaired in flies fed with AgNO_3_.

Apart from demelanization, assessment of few other biological processes revealed that 50 mg/L AgNP caused no interference with adult survival (Figure 2A), and metamorphosis remains unaffected (Figure 2C). Demelanized adult females lived the same median life span as the vehicle and citrate coating controls. On the other hand, flies exposed to 50 mg/L AgNO_3_ had a reduction in the median life span by twelve days. Based on this data, we determined that 50 mg/L is a nonlethal concentration for Drosophila. However, the number of progenies obtained from adult females raised in AgNP doped food were significantly lower than the controls especially during the peak egg-laying period, which is between 10–30 days (Figure 2B). Demelanized flies were less capable in their vertical movement ability, and this effect was evident starting from a very young age and sustained throughout (Figure 2E).

### AgNPs Interfere with Intracellular Copper Homeostasis

Current understanding on how silver is utilized inside the cell is almost non-existent. Some current mechanisms that are believed to be critical to AgNP toxicity include particle size, Ag^+^ release from the surface of the nanoparticle by oxidation, and interaction of AgNPs with biological molecules (e.g., proteins) upon entering the body [31]. AgNPs are also known to interact with thiol groups in proteins and promote their denaturation [32]. It was suggested that Ag interacts with proteins affiliated with maintaining cellular copper homeostasis [33]. Over all, the mechanism of AgNP toxicity is poorly elucidated until now. Cellular copper (Cu) transport and utilization, on the other hand, is a tightly regulated process consisting of proteins responsible for Cu uptake, distribution, and elimination [23], [28], [34]. We noticed an analogous but polar relationship exists between the Ag and Cu effects on the living system since excess Ag causes demelanization, which can be rescued by the addition of extra Cu in the diet so that the flies attain normal pigmentation (Figure S2). Finally, AgNP cause movement impairment similar to the effects of Cu starvation [23].

**Figure 3:**
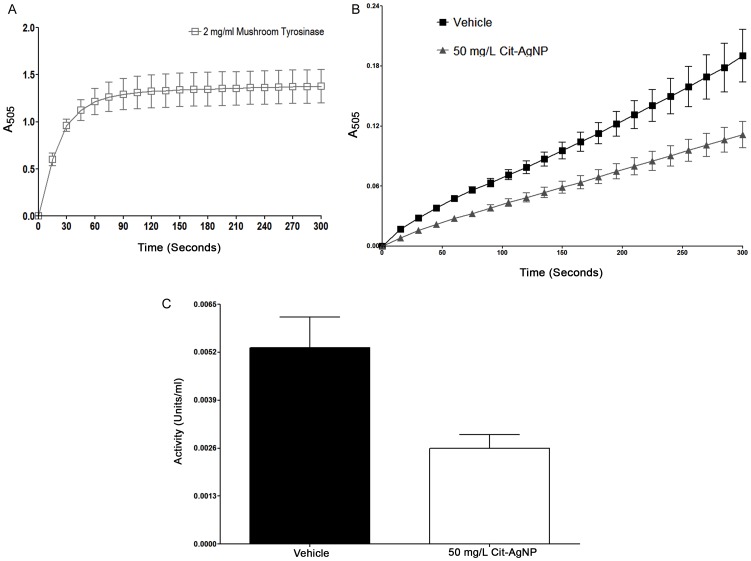
AgNP feeding negatively influence the tyrosinase enzyme activity. (A) Determination of purified mushroom tyrosinase activity (2 mg/ml) ensures a well-designed assay condition. The change in absorbance was recorded at a wavelength of 505 nm every 15 seconds for a total of five minutes. (B) Comparison of tyrosinase progress curves in fly extracts obtained from vehicle control and 50 mg/L Cit-AgNP fed flies showed that tyrosinase activity slows down significantly in AgNP fed flies compared to the control (C) Total tyrosinase activity in 50 mg/L Cit-AgNP fed flies was reduced to about 51% of the control value.

We therefore tested the impact of AgNP exposure on copper dependent enzymes such as tyrosinase and copper/zinc superoxide dismutase (Cu/ZnSOD) because these enzymes require Cu as a cofactor for their biological activity. Among them, tyrosinase is primarily required for synthesis of black melanin pigment [35]. The tyrosinase assay exploits the dopa oxidase branch of the pathway where L-dopa is converted to dopaquinone. Dopaquinone undergoes a Michael addition with MBTH to produce a dark pink pigment whose change in absorbance with time can be measured spectrophotometrically at a wavelength of 505 nm [28], [34]. It becomes quite evident from the progress curves that tyrosinase activity in AgNP fed flies increases at a slower rate in comparison to the vehicle control flies (Figure 3B). This resulted in lower tyrosinase activity in the AgNP fed flies with respect to the control. (Figure 3C).

**Figure 4:**
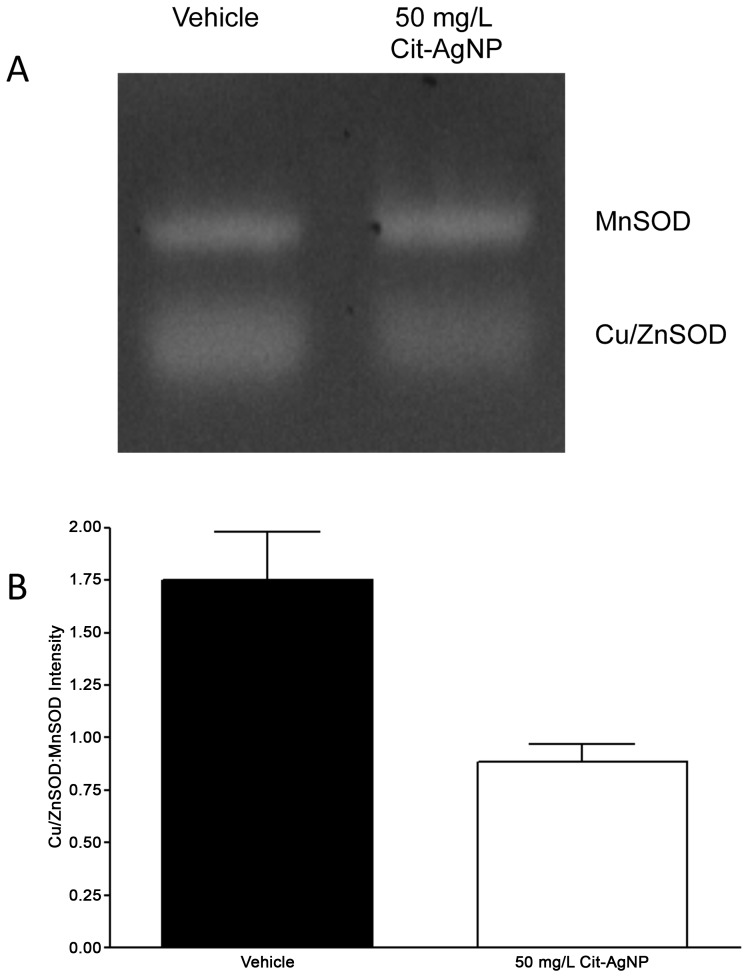
An in gel superoxide dismutase (SOD) activity determination in AgNP fed flies. (A) Electrophoretic separation of active Cu/ZnSOD and MnSOD enzymes and subsequent detection with the help of an electron donor shows two clear bands of enzyme activity. (B) Densitometric scanning of band intensities helped us to determine the activity ratios between Cu/ZnSOD and MnSOD enzymes, which suggests that the activity of the Cu/ZnSOD was significantly reduced in 50 mg/L Cit-AgNP fed flies with respect to the vehicle control. SOD activity analysis was performed in triplicate (n = 20 male flies/treatment).

Next, we measured the activity of Cu-ZnSOD in demelanized flies. An in-gel assay for superoxide dismutase (SOD) activity [32] has allowed the assessment of copper-zinc SOD (Cu-ZnSOD) activity with respect to intracellular manganese SOD (MnSOD) enzyme (Figure 4A). The Cu-ZnSOD/MnSOD ratio was reduced by ∼2 folds in demelanized flies compared to the control (Figure 4B). Thus, our results confirmed that, in the presence AgNPs, the activity of cellular copper dependent enzymes are reduced; on the contrary, the activity of MnSOD remains unchanged.

### Influence of AgNP on Cellular Copper Transporter

**Figure 5:**
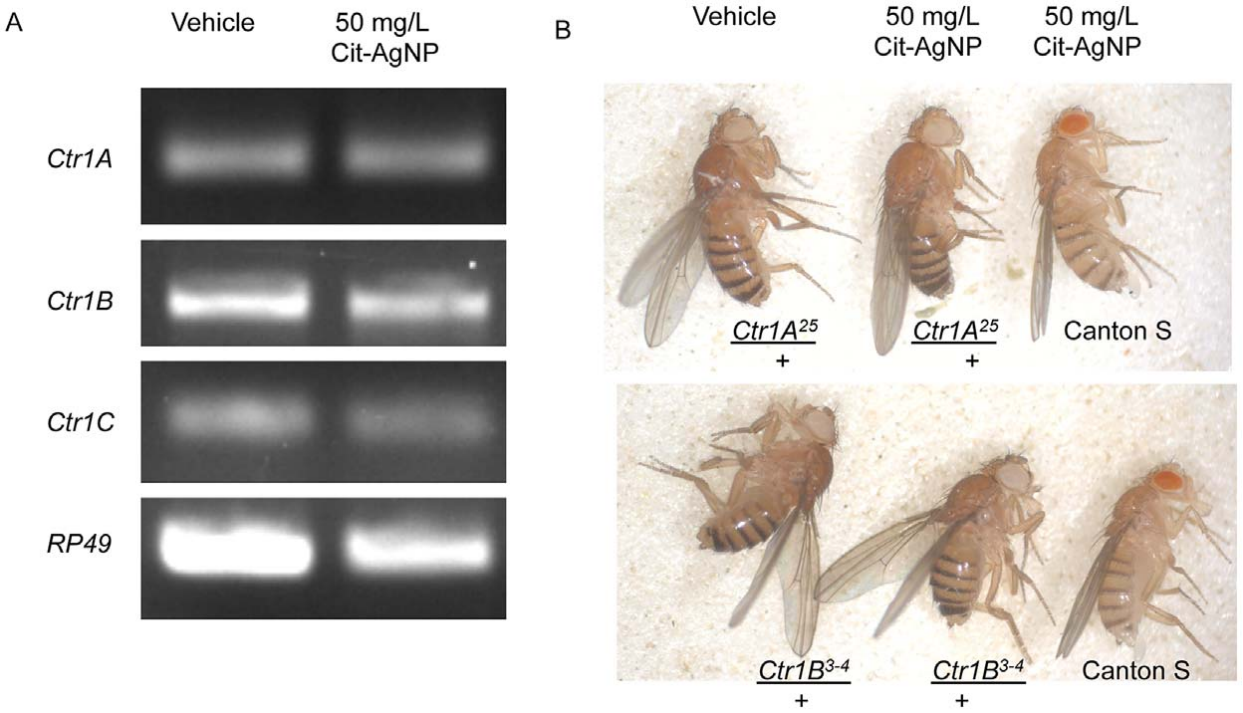
Copper transporters mutants are insensitive to demelanization. Comparative analysis of three *Ctr1* transcripts A, B and C expressions by RT-PCR showed no changes in mRNA expression following AgNP feeding with respect to the control flies suggesting that the copper transport machinery was not affected in the presence of Ag^+^. (B) Interestingly reduced synthesis of CTR1A and B proteins in *Ctr/+* heterozygotes makes them insensitive to demelanization effect, so their body color remains normal (darker abdominal stripes) as opposed to the AgNP fed fly which appears much lighter in body color. Therefore, intracellular copper transporters are required for transporting AgNPs into the cell.

Copper transporters (CTRs), a set of transmembrane proteins, are major vehicles for transporting copper inside the cell. In addition to CTRs, Cu can be transported inside the cell by other metal transporters as well [23]. Of the three copper transporters in Drosophila, *Ctr1B* mRNA expression is quite sensitive to changes in the intracellular Cu concentration; it increases when Cu^+^ is scarce, but is reduced when Cu^+^ is plentiful [23], [36]–[38]. Ag^+^ can be transported into the cells via CTRs [33] although it was not known whether *Ctr1* gene expression is regulated by AgNPs in a manner similar to Cu^+^. We therefore measured the mRNA transcription of the copper transporters *Ctr1A*, *Ctr1B*, and *Ctr1C* by RT-PCR, and observed no detectable variation occurs with respect to the *Ctr1* gene expression including *Ctr1B* in demelanized flies (Figure 5A).

Copper transport is an essential function, which is why *Ctr1* loss of function mutants failed to survive in mammals whereas in Drosophila *Ctr1B* homozygotes cannot survive under a Cu starvation situation [34], [39]. All *Ctr1/+* heterozygote adults with less Ctr1 RNA survive fine. To understand the involvement of Ctrs, we exposed the *Ctr1A^25^/+* and *Ctr1B^3-4^/+* mutant heterozygotes to 50 mg/L AgNPs the same way as the wild type flies and observed a very interesting effect. *Ctr1A^25^/+* and *Ctr1B^3-4^/+* heterozygotes have amazingly resisted the demelanization effect caused by AgNPs. Even though they were raised in 50 mg/L AgNP doped food, these flies appear completely normal in body color with no pigmentation defect (Figure 5B). This data suggests that AgNP works in conjunction with CTR proteins to cause the melanization effect, which is quite novel. Thus, we conclude that CTRs play an important role in AgNP induced demelanization effect and toxicity.

## Discussion and Conclusion

This study reports the effects of long-term exposure to AgNPs at a nonlethal concentration. We have been able to decode one biological mechanism whereby AgNPs interfere with the cellular Cu homeostasis to cause a consistent effect on cuticular melanization in insects. There are other biological processes that seem to be affected as well with long-term exposure to AgNPs like reduced female fertility and precipitous drop in movement ability as depicted. However, details on the mechanisms behind these two events remains to be further elucidated. Although the Drosophila system is not quite widely utilized in nanotoxicological studies, it is evident from this observation as well as from some recent ones [22], [40]–[41] that Drosophila offers a convenient and attractive biological system for evaluating the effects of Ag or any other nanoparticles because of the impressive response it displayed to nonlethal doses of AgNPs. Toxicity assessments subsequent to long-term exposure to nanoparticles appear to be more environmentally relevant [5] because the possibility of specific nanoparticles reaching up to an acutely toxic level in the atmosphere is infinitely small. Therefore, the overarching goal of this study was set up to evaluate all biological effects at a nonlethal concentration through long-term exposure to AgNPs.

**Figure 6:**
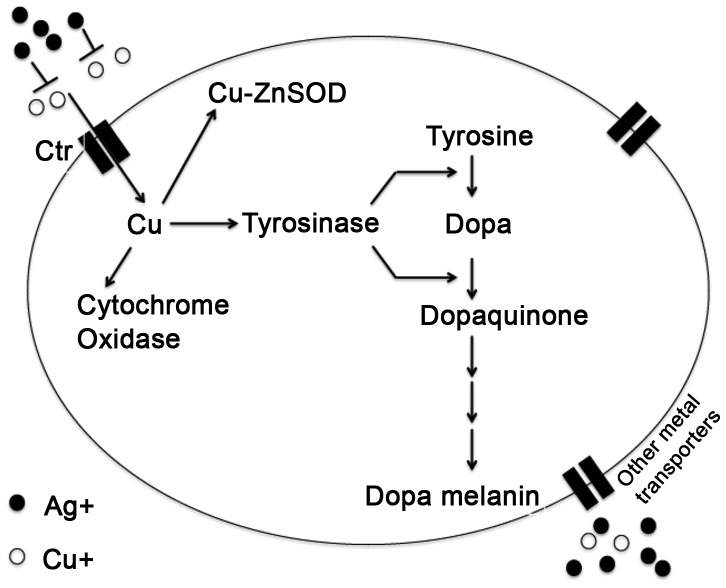
Schematic of silver and copper interaction in context of copper transport. Intracellular copper (Cu^+^) transport happens through conserved membrane bound copper transporter 1 (Ctr1) and also with the help of other metal transporters called general importers. *Drosophila* has three such CTR1 proteins, CTR1A, CTR1B, and CTR1C. Once inside the cell, Cu^+^ is utilized via three main branches to copper dependent proteins (e.g., tyrosinase), through copper chaperones to Cu-Zn superoxide dismutase (Cu/ZnSOD), and to cytochrome C oxidase in the mitochondria. Presence of excess Ag in the extracellular environment might inhibit the process of intracellular entry of Cu because Ag and Cu might be competing for the same copper transporters. Copper dependent tyrosinase is a dual action oxidase, which plays an essential role the conversion of tyrosine to dopa and dopa to dopaquinone, which is ultimately converted to melanin pigments. Thus, AgNPs can cause the demelanization effect through inhibiting Cu transport.

According to our data, consumption of AgNPs must be causing sequestration of Cu since the AA analysis suggests that overall Cu uptake was not limited in these animals (Figure 1C). Previous observations demonstrated that reduced synthesis of *Ctr1* mRNAs principally the *Ctr1B* gene leads to Cu starvation [34], [39], [42] and causing demelanization effect. However, none of the *Ctr1* mRNAs showed any changes in expression in the presence of AgNPs. On the contrary, Ctrs are actively required for AgNP action since reduced expression of both *Ctr1A* and *B* apparently makes the flies resistant to demelanization. This crucial experiment demonstrated the involvement of cellular copper transporters in this process. So, we propose a model to explain the effect of AgNPs, which suggests that when AgNPs are available in a high concentration, the uptake and availability of Cu inside the cell are influenced. We also prove that intracellular entry of Ag depends on CTRs, and thus presence of excess AgNP creates an intracellular Cu depletion effect through competitive inhibition (Figure 6). It is still possible that AgNPs might interfere with the general metal importers in addition to the CTRs, which might assist in Cu uptake from the immediate cellular environment [23].

Melanin synthesis is a highly conserved biochemical mechanism throughout the animal kingdom whereby melanin is made from the catecholamine precursors dopa and dopamine, which are synthesized from tyrosine [43]. Apparently, demelanization happens only when AgNPs are fed during the development but never happens if AgNPs are fed to adult animals. Therefore, AgNPs somehow interfere with the biosynthesis of melanin during development. In case of insects, pigments are incorporated into their hard exoskeleton during early adult life through a process called sclerotization [44]. For this reason, no demelanization has resulted when mature adult flies are exposed to AgNPs because pigment granules are already fixed in their cuticle through sclerotization. Cuticular pigmentation is an essential process in insect life because of its involvement with variety of diverse functions, which includes immunity, life span, and physiological and behavioral manifestations such as desiccation resistance [45] swarming, feeding, social dominance, mate preference and courtship display [46]. Therefore, the demelanization effect noticed in laboratory Drosophila at nontoxic concentration points to a situation where insect populations in the wild could be altered in a significant way if exposed to AgNPs.

Finally, the action of AgNPs on the activity of cellular Cu-ZnSOD enzyme fits well with the current dogma that most nanotoxic effects arise from excessive reactive oxygen species (ROS). Cu-ZnSOD is an essential enzyme in flies meant for dismutation of superoxide radicals mainly in the cytoplasm [47]. We demonstrate that the activity of this enzyme is decreased in the presence of AgNPs, although the reduction in Cu-ZnSOD activity is not enough to cause any negative effect on the viability of the fly. A recent observation claimed that exposure to AgNP increases the activity of Cu-ZnSOD and catalase enzymes [19] which seems counterintuitive because increased SOD and catalase activity should decrease cellular ROS production through dismutation. In that case, all downstream ROS mediated effects such as lipid peroxidation, and other hallmarks of oxidative stress should not be increased as claimed [48]. Our in-gel SOD activity should be able to clarify this point further. In context to changes in Cu-ZnSOD activity, it also brings a clear distinction of AgNP action on Cu-dependent enzymes only, since the activity of Mn dependent mitochondrial SOD remains unchanged. A recent observation reported that AgNPs influence the activity of selenium dependent proteins, namely thioredoxin reductase (TxR) that is involved with mitochondrial antioxidant defense system [49]. Thus, a picture is emerging slowly to understand the mechanism of AgNPs action on various biological processes.

## Supporting Information

Figure S1 (A) TEM of one batch of citrate-coated AgNP, (B) UV:Vis absorption spectrum (images courtesy of Stella Marinakos, Duke University).
(TIF)

Figure S2 Excess Cu prevents AgNP induced demelanization effect. AgNP doped food was mixed with Cu in indicated concentrations. We found that addition of Cu prevents the demelanization effect since the flies appeared with regular body pigmentation.
(TIF)
