# Supplementary figures and images for: Mechanism of Silver Nanoparticles Action on Insect Pigmentation Reveals Intervention of Copper Homeostasis
Source: PLoS One. 2013 Jan 7;8(1):e53186. doi: 10.1371/journal.pone.0053186 (PMC3538783; doi:10.1371/journal.pone.0053186)

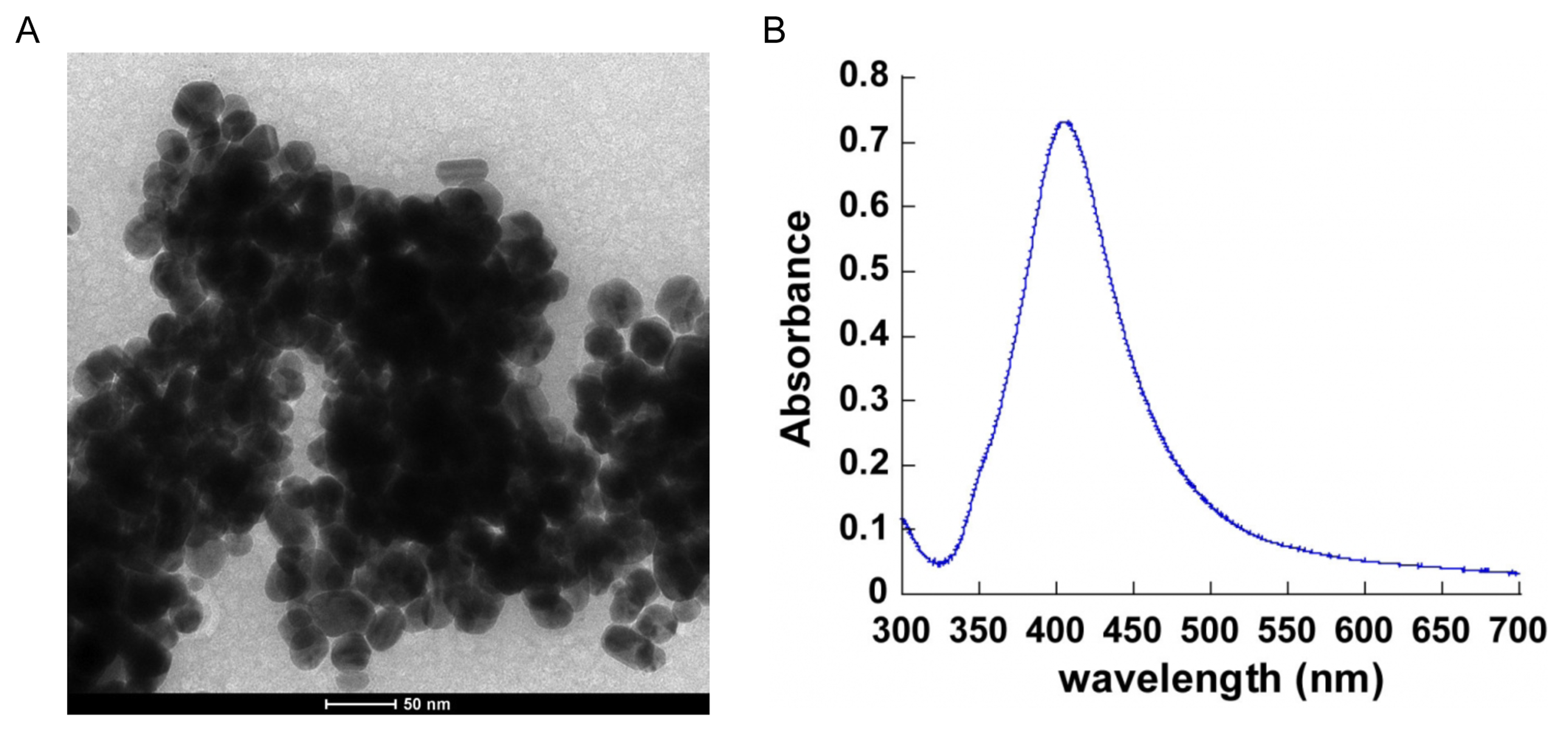

Supplement: Figure S1 — (A) TEM of one batch of citrate-coated AgNP, (B) UV:Vis absorption spectrum (images courtesy of Stella Marinakos, Duke University). (TIF) [file pone.0053186.s001.tif]

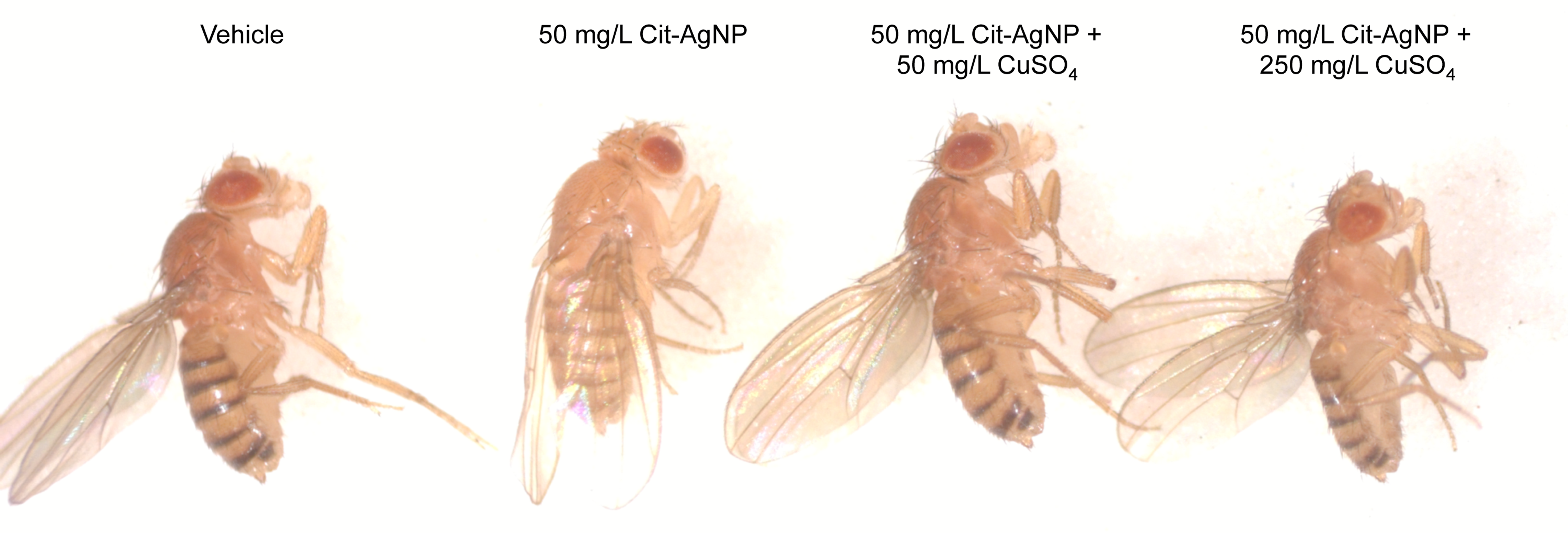

Supplement: Figure S2 — Excess Cu prevents AgNP induced demelanization effect. AgNP doped food was mixed with Cu in indicated concentrations. We found that addition of Cu prevents the demelanization effect since the flies appeared with regular body pigmentation. (TIF) [file pone.0053186.s002.tif]
